# Seasonal Microbial Conditions of Locally Made Yoghurt (Shalom) Marketed in Some Regions of Cameroon

**DOI:** 10.1155/2017/5839278

**Published:** 2017-12-20

**Authors:** Lamye Glory Moh, Lunga Paul Keilah, Pamo Tedonkeng Etienne, Kuiate Jules-Roger

**Affiliations:** ^1^Department of Biochemistry, University of Dschang, Dschang, Cameroon; ^2^Department of Biochemistry, University of Yaounde 1, Yaounde, Cameroon; ^3^Department of Animal Production, University of Dschang, Dschang, Cameroon

## Abstract

The microbial conditions of locally made yoghurt (shalom) marketed in three areas of Cameroon were evaluated during the dry and rainy seasons alongside three commercial brands. A total of ninety-six samples were collected and the microbial conditions were based on total aerobic bacteria (TEB), coliforms, yeasts, and moulds counts as well as the identification of coliforms and yeasts using identification kits. Generally, there was a significant increase (*p* ≤ 0.05) in total aerobic and coliform counts (especially samples from Bamenda), but a decrease in yeast and mould counts of the same samples during the rainy season when compared to those obtained during the dry season. These counts were mostly greater than the recommended standards. Twenty-one Enterobacteriaceae species belonging to 15 genera were identified from 72 bacterial isolates previously considered as all coliforms.* Pantoea *sp. (27.77%) was highly represented, found in 41% (dry season) and 50% (rainy season) of samples. In addition, sixteen yeast species belonging to 8 genera were equally identified from 55 yeast isolates and* Candida *sp. (76.36%) was the most represented. This result suggests that unhygienic practices during production, ignorance, warmer weather, duration of selling, and inadequate refrigeration are the principal causes of higher levels of contamination and unsafe yoghurts.

## 1. Introduction

Yoghurt consumption has become very popular in Cameroon ever since the production of locally made yoghurt started. Yoghurt in itself is a very nutritious diet [1] for people across all age groups. Yoghurt quality varies from one producer to another as there is no well-described standard for its production. In Cameroon, it is generally produced with leftover “shalom yaourt” or any commercial brand of yoghurt (Camlait or Dolait) for fermentation [2]. Consumers are becoming more inquisitive about the quality of these fermented products due to episodes of diarrhoea they experienced at times. Its high and easily assimilable nutritive value provides a suitable environment for microbial contamination, proliferation, and spoilage. Microbial contamination can lead to food poisoning outbreaks and unsatisfactory products [3] and this is an enormous economic problem worldwide. Unsafe food is still an important threat in most developing countries, especially in Africa [4, 5]. Microbial contamination and foodborne microbial diseases constitute a large and growing public health concern. In fact, most countries with case-reporting systems have documented significant increases over the past few decades in foodborne microbial diseases incidence [6]. Milk is a highly nutritious food that serves as an excellent growth medium for a wide range of microorganisms [7]. Through microbial activity alone, approximately one-fourth of the world's food supply is lost [8]. Undesirable microbes that can cause spoilage of dairy products include Gram-negative psychrotrophs, coliforms, lactic acid bacteria, yeasts, and moulds. For this reason, increased emphasis should be placed on the microbiological examination of dairy products.

Food safety challenges in Africa include unsafe water and poor environmental hygiene, weak foodborne disease surveillance, inability of small and medium scale producers to provide safe food, outdated food regulations, and inadequate law enforcement, as well as insufficient cooperation among stakeholders. The World Health Organization requires that small scale dairy processing plants in the developing countries urgently comply with the Codex Alimentarius principles [5]. Few African countries have enacted foodborne disease surveillance systems; in Cameroon regulations concerning hygienic control of dairy products have been issued, but they are rarely enforced, and the hygienic condition of the milk chain is not sufficiently controlled (Njomaha, personal communication). Enterobacteriaceae and coliform bacteria within this family represent two of the most common groups of indicator organism used by the food industry [9]. Enterobacteriaceae, a large and heterogeneous family of Gram-negative bacteria, may constitute health hazards to the consumers [10] if present in yoghurt. They are useful indicators of overall GMP, but not necessarily faecal contamination [9]. The presence of indicators organisms usually indicates that a potential problem or failure in the process has occurred, whereas their absence in food provides a degree of assurance that the hygiene and food manufacturing process has been carried out appropriately. The most important index of microbiological quality is total bacterial counts, coliforms, yeasts, and moulds and detection of specific pathogens and their toxins as recorded by Kwee et al. [10].* E. coli*, a coliform, is considered as normal flora of intestinal tracts of humans and animals. They have been used as indicator organisms for bacteriological quality of milk and its products [11]. Generally the presence of higher number of coliforms indicates heavy contamination caused by unsanitary conditions and poor production [12]. Although bacteria can be food spoilage organisms, yeasts and filamentous fungi are often involved in the deterioration of yoghurts [13, 14]. They are a major cause of yoghurt spoilage [15, 16] and their growth is favoured by the low pH of yoghurt [13, 17]. The presence of yeasts and moulds in milk and dairy products is undesirable even in small amounts due to the resulting objectionable changes that lower the products quality [18]. They are responsible for off-flavours, loss of texture quality due to gas production, and package swelling and shrinkage [19]. More so, moulds and yeasts growing in yoghurt utilize some of the acid and produce a corresponding decrease in the acidity, making the food environment more susceptible for proteolysis and putrefaction by bacteria [15, 20].

A lot of work has been done on the hygienic quality of yoghurt or locally made yoghurt in most part of the world [21–26]. Most of them concluded that the locally made types were of lower hygienic quality when compared to the commercial brands. In Cameroon, little or nothing has been done on the identification of these microbes, and no published studies exist on microbial quality of yoghurts produced and sold in Northwest and West Regions (Dschang and Bafoussam) of Cameroon. Even when an attempt is done in some of the above places or other regions of the country on dairy products, it often ends at the level of colony count which can be misleading at times without identification. Cameroon has two seasons which are the rainy and dry seasons. The rainy season begins in March and ends around October depending on the part of the country, while the dry season begins in October/November and ends in March. Most streets, especially in the rural areas, are dirt roads, very dry, and dusty during dry season and become very muddy during the rainy season; thus, this study was carried out during the two seasons. Research in the field of safety/quality evaluation of market yoghurt/shalom is essential to create awareness among common people about the existing situation and protect the consumer's health and rights. Therefore, this study was conducted to evaluate the microbial quality of locally made yoghurts (shalom) available in some regions of Cameroon.

## 2. Materials and Methods

### 2.1. Collection of Samples

Samples of locally made yoghurts were collected from 15 (dry seasons) and 14 (rainy seasons) producers at three different occasions from some Regions (Bamenda, Bafoussam, and Dschang) of Cameroon from 2012 to 2013. This gave a total of eighty-seven samples: forty-five and forty-two during the dry (November–January) and rainy (May–early August) seasons, respectively. Concurrently, three commercial brands of yoghurt, (BB), (AA), and (CC), available in Cameroon were equally collected on the same day from a well-known sale point in Dschang, giving an overall sample size of ninety-six. Samples were collected in sterile and labeled containers and transported under aseptic conditions in an ice packed container, at 4–7°C to the Laboratory of Microbiology and Antimicrobial Substances, Faculty of Science, University of Dschang.

### 2.2. Microbiological Analysis

#### 2.2.1. Preparation of Materials

All media were obtained in dehydrated forms and prepared according to the manufacturer's instructions. Glassware such as Petri-dishes, test tubes, pipettes, flasks, and bottles was sterilized in a hot oven at 170°C for two hours, whereas distilled water was sterilized by autoclaving for 15 min at 121°C [27].

#### 2.2.2. Preparation of Serial Dilutions

This was done according to APHA [28] in which 1 ml of yoghurt from a homogenous sample was serially diluted into 9 mL of sterile distilled water to prepare eightfold dilutions from 10^−1^ to 10^−8^. 50 *μ*l of diluted samples were spread over prepared dried plates with different media.

#### 2.2.3. Enumeration of Total Aerobic Bacteria (TEB)

Nutrient agar (Oxoid) was used to determine the total aerobic bacterial count [29] and appropriate dilutions were pour-plated. The cultured plates were incubated aerobically at 37°C for 24 h. TEB was counted after the colonies were evaluated [30].

#### 2.2.4. Enumeration of Coliform Bacteria

MacConkey agar (Oxoid) was used to determine the coliform count [29, 31]. The cultured plates were incubated aerobically at 37°C for 24 h after pour plating of the appropriate dilutions. The colonies were evaluated and counted at the end of the incubation [30].

#### 2.2.5. Enumeration of Yeast and Moulds

Sabouraud Dextrose agar (Oxoid) (supplemented with 0.5 g/l chloramphenicol) was used to determine yeast and mould counts [32]. After the pour-plated plates were incubated aerobically at 25°C for 3–5 days, the developed colonies were evaluated and counted.

### 2.3. Isolation and Identification of Microorganisms

The distinguished colonies on the incubated plates were picked and purified by repeated subculturing done by streaking on the appropriate media with a sterile loop (the strategy consisted of picking 1 colony to represent every visibly different morphology on each plate) using the streak method. Purified colonies were prepared in their respective broth: Mueller Hinton broth for coliforms and Sabouraud Dextrose broth for yeast and moulds. From these preparations, 0.5 ml of each was pipitted into 0.5 ml of glycerol and stored in a freezer at −5°C awaiting identification. All the bacterial cultures were subcultured prior to their use in further experiments and the obtained fresh cultures were used for biochemical tests.

By microscopic observation of each culture following incubation, the purity of isolates was confirmed and preliminary identifications were done according to Bergey's Manual [33, 34]. Proper identification to species level was carried out on the basis of biochemical tests with API 20E (for identification of Enterobacteriaceae and other nonfastidious Gram-negative rods) and API 20 C AUX (for the identification of yeast) (bioMerieux, Marcy l'Etoile, France) according to the instructions of the manufacturer.

### 2.4. Statistical Analysis

Data were subjected to the one-way analysis of variance (ANOVA), and differences between samples at *p* ≤ 0.05 were determined by Waller Duncan test using the Statistical Package for the Social Sciences (SPSS) version 11.0. The results were presented as an average of the logarithm (log_10_) of colony forming unit (cfu)/ml (log 10 cfu/ml) in the samples as mean ± SD of the replicates.

## 3. Results and Discussion

### 3.1. Total Aerobic Bacterial Counts

The total aerobic bacterial counts of all the samples (Table 1) during the dry season in Cameroon (November–January) were very high when compared to other results. Samples from Dschang had bacterial counts of 9.28 ± 0.00 to 11.63 ± 0.10; Bamenda, 8.80 ± 0.06 to 11.78 ± 0.02; Bafoussam 11.18 ± 0.04 to 11.36 ± 0.14; and commercial brands 9.54 ± 0.05 to 11.48 ± 0.11 (log 10 cfu/ml). Samples from Bafoussam had the highest total bacterial count (with no variation within 75% of samples), followed by those from Bamenda, commercial brands, and Dschang. As shown in Table 2, total aerobic bacterial counts varied significantly (*p* ≤ 0.05) with each other as well as the commercial brands during the rainy season. These counts were still very high when compared to 6.77 obtained by Al-Tahiri [35] with yoghurts produced by modern dairies in Jordan and 7.86 from Younus et al. [21] obtained from dahi (locally made yoghurt in Pakistan and India).

During the rainy season, samples from Dschang had bacterial counts of 8.70 ± 0.09 to 11.55 ± 0.06; Bamenda, 9.17 ± 0.13 to 11.73 ± 0.01; Bafoussam, 11.02 ± 0.08 to 11.75 ± 0.03; and commercial brands, 10.34 ± 0.12 to 11.34 ± 0.06 (log 10 cfu/ml). On average, bacterial count of all samples was significantly higher (*p* ≤ 0.05) than the commercial brands. Samples from Bafoussam had the highest aerobic bacterial count, followed by those from Dschang and then Bamenda (Table 2). However, 25% of samples from Bamenda and 50% of samples from Dschang had total bacterial counts less than that of the least commercial brand. Generally, there was a significant increase (*p* ≤ 0.05) in total count during the rainy season when compared to those obtained during the dry season (especially samples from Bamenda). This could be due to the low turnover of yoghurt during this season since a considerable number of people ignorantly consume it just to quench taste, regardless of the nutritional value. Consequently, temperature fluctuation resulting from longer selling/storage time may offer a favourable environment for the multiplication of these bacteria.

High bacterial count is also expected because of the presence of starter cultures, which are mainly lactic acid bacteria. The standard aerobic bacterial count is 10^6^–10^7^ cfu/ml [36, 37], corresponding to 6-7 in log 10 cfu/ml. Thus, the results of this study showed that total aerobic bacterial count in all samples was very high relative to the standard values. Very high count however is used as an indication of postpasteurisation contamination [38], due to inadequate hygienic measures during production. In most foods, the total bacterial count is often an indication for the sanitary quality, safety, and utility of foods. It may reflect the conditions under which the product is manufactured such as contamination of raw materials and ingredients, the effectiveness of processing, and the sanitary conditions of equipment and utensils at the processing plants [11]. Storage in unhygienic conditions and prolonged storage time can also contribute to this [39]. This signals the paramount need for a sensitization of yoghurt producers involved in this study as per the hygienic conditions of their production processes.

### 3.2. Coliform Counts

A majority of the samples had coliform counts higher than 10^2^ cfu/ml (2 in log 10 cfu/ml) (Table 1) which is the maximum determined in most of the international standards (Kucukoner and Tarakci, 2003). The coliform count of samples from Dschang during the dry season ranged from 4.29 ± 0.20 to 5.12 ± 0.09; Bamenda, 3.30 ± 0.00 to 4.82 ± 0.16; Bafoussam, 1.25 ± 2.18 to 6.90 ± 0.08; and commercial brand, 0.00 ± 0.00 to 4.81 ± 0.05 (log 10 cfu/ml). Coliform count of samples varied significantly (*p* ≤ 0.05) from each other. Samples from Dschang (66.66%), Bamenda (62.50%), and Bafoussam (50%) were significantly lower (*p* ≤ 0.05) than the commercial brands. Generally, during the rainy season counts were still greater than 10^2^ cfu/ml (Table 2). Samples from Dschang had coliform counts from 4.49 ± 0.35 to 5.55 ± 0.07; Bamenda, 3.76 ± 0.40 to 5.51 ± 0.03; Bafoussam, 0.00 ± 0.00 to 5.10 ± 0.02; and commercial brands, 0.00 ± 0.00 to 4.85 ± 0.04 (log 10 cfu/ml). Coliform counts generally increased in 47.05% and decreased in 41.17% during the rainy season. Like in total bacterial count, there was a significant increase (*p* ≤ 0.05) in coliform count during the rainy season when compared to those obtained during the dry season in samples from Bamenda, while those from Bafoussam decreased. Interestingly, sample BB (commercial) was void of coliforms during both seasons as well as C from Bafoussam during the rainy season (Tables 1 and 2).

These results are in line with the work of Moreira et al. [40] who reported that warmer weather and inadequate refrigeration are the principal causes of higher levels of contamination. Coliforms detection or enumerations are often used as parameters for evaluating yoghurt quality in different countries [12, 41, 42]. It is an indicator of poor hygiene, inadequate processing, or postprocessing contamination in yoghurt as established and recommended by public health authorities worldwide, used as indicator organisms for bacteriological quality of milk and its products [11]. The high levels of coliform counts (3.30 ± 0.00 to 6.90 ± 0.08 log 10 cfu/ml) in both the locally made varieties and even the commercial brands (except BB) might indicate a low level of hygiene and improper sanitation during/after the manufacturing process [43] or insufficient preheating during production. It also shows negligence in sanitary measures especially the commercial brands which are regarded as quality-controlled products. Meanwhile, the absence of coliforms in some samples as mentioned above is an indication of Good Manufacturing Practices (GMP) employed by the producers and retailers [25, 44]. Coliforms are not supposed to be present in yoghurt in such high levels because of pasteurisation and controlled hygienic procedures [32]. The presence of coliforms in these yoghurts could pose an adverse effect in consumers' health and suggests negligence on the part of the producers or the yoghurt vendors as well as quality controllers. According to the standard stipulated by the National Agency of Food and Drug Administration Control (NAFDAC),* E. coli *and coliforms generally must not be detectable in any 100 ml of yoghurt sample [45]. Other probabilities of contamination can be from contaminated water source and equipment used or due to contamination at storage and display/sale outlet [46].

### 3.3. Coliform Species and Other Enterobacteriaceae

A total of seventy-two (72) bacterial isolates (40 during the dry season (I) and 32 during the rainy season (II)) were identified to specie level from ninety-six (96) yoghurt samples (Table 3). They were distributed as follows: 27 (14 I and 13 II), 14 (10 I and 4 II), 25 (13 I and 12 II), and 6 (3 I and 3 II) from Bamenda, Dschang, Bafoussam, and commercial brands, respectively. Twenty-one (21) bacteria species belonging to 15 genera were identified, with the number of occurrences indicated in parentheses:* Pantoea *sp.* 1 *(15)*, Pantoea *sp.* 3* (3)*, Pantoea *sp.* 4* (2)*, Klebsiella pneumonia *ssp.* pneumonia *(11)*, Klebsiella oxytoca *(1)*, Providencia stuartii *(5)*, Providencia rettgeri* (2)*, Providencia alcalifaciens/rustigianii *(1)*, Shigella *sp. (7)*, Enterobacter aerogenes* (3)*, Enterobacter cloacae *(3),* Serratia plymuthica *(5)*, Escherichia coli 1 *(3),* Burkholderia cepacia* (3),* Citrobacter freundii *(2),* Pseudomonas oryzihabitans* (2),* Moellerella wisconsensis* (2),* Raoultella ornithinolytica* (1)*, Pasteurella pneumotropica/Mannheimia haemolytica* (1)*, Ochrobactrum anthropi *(1)*, and Proteus penneri* (1) (Table 3).

Generally, the percentage of isolates was higher during the dry season (55.55%) when compared to those of the rainy season (44.44%).* Pantoea *sp. (27.77%) was the highest represented species with 21.42%, 20.00%, 46.13%, and 0.00% (dry season) as well as 38.46%, 0.00%, 25.00%, and 33.33% (rainy season) from Bamenda, Dschang, Bafoussam, and commercial brands, respectively. It was present in all the batches (except commercial samples during the dry season and Dschang during the rainy season) and found in 7 out of 17 (dry season) and 8 out of 16 (rainy season) samples. This was followed by* Klebsiella *sp. (16.66%), absent in Bamenda throughout but occupying 20%, 23.07%, and 33.33% (dry season) when compared to 75.00%, 8.33%, and 33.33% (rainy season) isolates from Dschang, Bafoussam, and commercial brands, respectively. The third candidate was* Providencia* sp. (11.11%), absent in the commercial samples throughout the season with 21.48%, 10.00%, and 0.00% (dry season) as well as 15.38%, 0.00%, and 8.33% (rainy season) from Bamenda, Dschang, and Bafoussam, respectively. The fourth was* Shigella *sp. (9.72%), absent in Dschang during both seasons, Bafoussam, and commercial brands (rainy season) but present in Bamenda during the two seasons. Notwithstanding, the lowest frequency of occurrence (1.38%) was recorded by* Raoultella ornithinolytica, Pasteurella pneumotropica/Mannheimia haemolytica, Ochrobactrum anthropi, and Proteus penneri *as shown in Table 3.

It can be observed in Table 4 that some of these bacteria were present only in one of the places or yoghurt brand, for example, species like* Pantoea *sp.* 3, Pantoea *sp.* 4, Raoultella ornithinolytica and Providencia alcalifaciens/rustigianii *(Bamenda),* Ochrobactrum anthropi, Providencia rettgeri, and Pasteurella pneumotropica/Mannheimia haemolytica* (Dschang in dry season) as well as* Klebsiella oxytoca*,* Citrobacter freundii,* and* Escherichia coli 1* in samples from Bafoussam during the rainy season (II). Also, the specie* Proteus penneri* was only observed in the commercial sample (CC). The rest of the species were present in 2 or 3 places and in one season or the other (Table 4).

There were differences in the bacteriological load of batches of yoghurt samples assessed. This together with the isolation of indicator organisms shows failure of GMP in industries and local producers that manufactured these yoghurts from which they were isolated [47]. The genus* Pantoea *includes several species of which others can cause disease in humans such as tumors [48, 49]. However,* E. coli, *an index organism, indicates the presence of other pathogenic microorganisms and has been linked to diarrhoeal diseases, urethrocystitis, prostatitis, and pyelonephritis [23]. More so, Enteropathogenic* E. coli* have also been incriminated as a potential food poisoning agent and are associated with infantile diarrhoea and gastroenteritis in adults.* E. coli* might had entered into some of these yoghurt samples through water used in production, unhygienic hawking habits, and storage environment and not necessarily failure of GMP. Meanwhile,* Klebsiella *sp., another coliform, may be an indicator of product contamination through faecal contaminated water or raw materials [50].

Coliforms have been related to bacterial pneumonia cases more severe than those produced by* Streptococcus pneumonia *and urinary tract infection. This is the case of* K. pneumoniae *and* K. oxytoca *which are opportunistic pathogens and have been linked over the years as the main cause of septicaemia, pneumonia, urinary tract infections, and soft tissue infections [51, 52].* Burkholderia cepacia *is also known for pneumonia or bacterial infections that occur in patients with impaired immune systems or chronic lung disease, particularly cystic fibrosis (CF). Infection with* Shigella *sp. is normally limited to the distal ileum and colon, and common symptoms include diarrhoea, fever, nausea, vomiting, stomach cramps, and flatulence. In cases of* Shigella*-associated dysentery, the epithelial cells of the intestinal mucosa in the caecum and rectum are destroyed.* Shigella *has also been implicated as one of the causes of reactive arthritis. Apart from that,* E. cloacae *and* C. freundii *are associated with illnesses such as necrotizing enterocolitis, diarrhoea, meningitis, urinary tract infections, intra-abdominal and ophthalmic infections, and septicaemia [53–55]. Generally, the presence of* Enterobacter *sp. in yoghurt or other foods may be caused by poor environmental conditions due to dust and contaminated water used in production. These species have been known to be inhabitants of dairy products [50]. Meanwhile,* Citrobacter *sp. has been shown to carry various virulence determinants found in other pathogens.* Providencia alcalifaciens*, a food poisoning agent, causes diarrhoea [56] especially in children and* Providencia stuartii* is also linked to infective endocarditis [57]. In addition,* Providencia rettgeri* is the cause of ocular infections, including keratitis, conjunctivitis, and endophthalmitis [58] as well as travelers' diarrhoea.* P. alcalifaciens*,* P. rettgeri*, and* P. stuartii* have generally been implicated in gastroenteritis. Applebaum and Campbell [59] reported that* Ochrobactrum anthropi *was responsible for an infection in humans known as pancreatic abscess. In addition,* Raoultella ornithinolytica* has been known to cause enteric fever-like syndrome [60], giant renal cyst leading to colic obstruction [61], and* R. ornithinolytica *bacteremia [62].* Pseudomonas* is found in soil, water, plants, and animal and is present in small percentage in the normal intestinal flora and on the human skin [63]. Lastly* P. penneri* has the ability to cause major infectious diseases and nosocomial outbreaks [64] and carries similar pathogenic determinants like* P. mirabilis* and* P. vulgaris* [65]. It usually infects urinary tract, blood, neck, and ankle [65, 66]. Thus, these yoghurts predispose their consumers to a vast array of diseases whose causative agents are supposed to be susceptible to pasteurisation. However, their presence in postpasteurised yoghurt may be as a result of inadequate heating process, the use of contaminated water, postproduction contamination, and the presence of poor sanitary behaviours during packaging and storage conditions at the production areas as well as unhygienic hawking habits.

### 3.4. Yeast Counts

Generally, there was no significant difference (*p* > 0.05) in yeast counts amongst the samples during the dry season (Table 1). The present data show that 87.5% and 66.66% of samples from Bamenda and commercial brands had yeast, while it was present in 100% of samples from Dschang and Bafoussam during the dry season. Samples from Dschang had counts from 3.73 ± 3.23 to 4.97 ± 0.11; Bamenda 0.00 ± 0.00 to 4.94 ± 0.15; Bafoussam, 4.02 ± 0.10 to 4.63 ± 0.13; and commercial brands, 0.00 ± 0.00 to 4.84 ± 0.49 (log 10 cfu/ml). Samples collected from all the localities had yeast count (4.02 ± 0.21 to 4.97 ± 0.11 (log 10 cfu/ml)) higher than 3log⁡10 cfu/ml which is the international standards [35, 37, 67]. There was a decrease in yeast counts during the rainy season (Table 2) in samples from Bamenda and an increase in those from Bafoussam when compared to most of the samples collected during the dry season (Table 1). Still, 87.5% and 66.66% of samples from Bamenda and commercial brands had yeast, as well as 100% of samples from Dschang and Bafoussam. The yeast count of samples from Dschang was from 4.05 ± 0.18 to 4.87 ± 0.10; Bamenda, 0.00 ± 0.00 to 4.41 ± 0.06; Bafoussam, 4.49 ± 0.31 to 5.05 ± 0.02; and commercial brands, 0.00 ± 0.00 to 4.77 ± 0.17 (log 10 cfu/ml). Throughout the seasons, samples from Bafoussam had the highest yeast counts, followed by those from Dschang, Bamenda, and commercial brands the least. The higher yeast count during the dry season might reflect the ability of more yeast to grow during warmer weather [39], increased species diversity, and alteration in microbial flora leading to higher levels of contamination. This explains why there was an increase in yeast count during the dry season when compared to yoghurt sold in the rainy season.

High counts of yeast and mould have also been reported in yoghurts [23, 68–70]. Though higher than the international standard in most cases, yeast counts in this study corroborated those reported in Australia [71, 72], Nigeria [73], and Egypt [74]. However, examples of yeast occurrences in yoghurts with more than 6log⁡10 cfu/ml [75, 76] and 3log⁡10 cfu/ml or lesser have also been recorded from various countries such as UK, Canada, USA, and the Netherlands [77–79]. The high levels of moulds and yeast obtained in this study are attributed to poor handling and production [20, 66, 80]. Certain yeasts play an important role in the spoilage of fermented products. Since milk is pasteurised before yoghurt production, the presence of yeasts in yoghurt is caused by inappropriate pasteurisation and/or recontamination processes during manufacture [75].

### 3.5. Mould Counts

The control samples were void of moulds, while 100%, 87.5%, and 50% of samples from Dschang, Bamenda, and Bafoussam had moulds, respectively, during the dry season (Table 1). Yoghurt samples from Dschang and Bamenda had mould counts of 0.00 ± 0.00 to 4.26 ± 0.11 and Bafoussam 2.53 ± 2.20 to 3.50 ± 0.17 (log 10 cfu/ml). The control samples were still void of moulds during the rainy season with a reduction in the spread and count of moulds in all the regions. In this season, 50%, 57.14%, and 25% of samples from Dschang, Bamenda, and Bafoussam had moulds, respectively. A maximum of 2log⁡10 cfu/ml of mould is allowed in yoghurt [81]. Yoghurts having initial mould counts > 2log⁡10 cfu/ml tend to spoil quickly and may even spoil before refrigeration [40]. This standard was not met in 27.78% (Table 1) and 29.41% (Table 2) of all samples. There was a clear association between levels of yeast and moulds contamination and season, as counts were higher during the dry season and lower during the rainy season. The presence of high yeast and mould counts in examined yoghurt samples may also indicate inefficient preheating process during manufacturing, using unsatisfactory sterilized plastic cups in packing or inefficient chilling on storage [82]. However, it could as well be attributed to contamination from air and the old yoghurt or commercial yoghurt used as starter culture during production. Mould and yeast contamination causes deterioration and influences the biochemical characters and flavour of the product and its appearance is commercially undesirable and often results in downgrading of the product. Spoilage becomes evident when yeast populations reach 5 to 6 (log 10 cfu/ml) and is first recognized as a swelling of the yoghurt package due to gas production by yeast fermentation. The yoghurt acquires a yeasty, fermented odour and flavour and a gassy appearance which eventually ruptures; colonies of yeasts on the undersurface of the package lid can be seen at times [13].

### 3.6. Yeast Species

Fifty-five yeast isolates (25 during the dry season (I) and 30 during the rainy season (II)) were identified to specie level from ninety-six yoghurt samples (Table 5). They were distributed as follows: 28 (11 I and 17 II), 9 (7 I and 2 II), 12 (5 I and 7 II), and 6 (2 I and 4 II) from Bamenda, Dschang, Bafoussam, and commercial brands, respectively. Sixteen yeast species belonging to 8 genera were identified, with the number of occurrences indicated in parentheses:* Candida zeylanoides *(15),* Candida kruzei/inconspicua* (14)*, Candida dubliniensis* (6),* Candida lusitaniae* (3),* Candida boidinii* (2),* Candida albicans 1 *(1),* Candida albicans 2 *(1),* Trichosporon asahii *(3),* Stephanoascus ciferrii *(2),* Kodamaea ohmeri *(2),* Rhodotorula mucilaginosa 1 *(1),* Rhodotorula mucilaginosa 2 *(1),* Pichia angusta *(1),* Cryptococcus laurentii *(1),* Cryptococcus humicola *(1), and* Kloeckera *sp. (1) (Table 5).

Generally, the percentage of isolates was higher during the rainy season (54.54%) when compared to that of the dry season (45.45%).* Candida *sp. was the highest among the isolates (76.36%), with the highest percentage contributed by* Candida zeylanoides *(27.27%)*, Candida kruzei/inconspicua* (25.45%), and* Candida dubliniensis* (10.90%). Other species that were detected in lower percentages were* Candida lusitaniae* (5.45%),* Candida boidinii* (3.63%),* Candida albicans *1 (1.81%), and* Candida albicans *2 (1.81%).* Candida zeylanoides *occupying the highest percentage was absent in the commercial samples (dry and rainy seasons) and those from Dschang and Bafoussam (dry season). It was mostly represented during the dry season with 27.27% (present in all samples) and 42.85% and 20.00% in samples from Bamenda, Dschang, and Bafoussam, respectively, though it was higher in Bamenda (45.45%) during the rainy season.* Candida kruzei/inconspicua *was absent in Dschang but represented 45.45%, 40.00%, and 50.00% (dry season) as compared to 23.52%, 14.28%, and 25.00% (rainy season) in samples from Bamenda, Bafoussam, and commercial brands, respectively. On the other hand,* Candida dubliniensis* either present during the dry (Bamenda) or rainy season (Bafoussam) or even during both seasons (commercial brands) having the highest percentage in Bafoussam (28.57%). With the exception of* Candida lusitaniae* which was found in 3 of the 4 groups of samples (Dschang, Bafoussam, and commercial brand), the rest were present either in just one or two of these groups or in just one of the seasons (Table 6). There was no association between total yeast count and number of species found but the diversity was greater during the rainy season when compared to those of the dry season. This could lead to increased mutualism in the breakdown/utilization of the food substrate and thus enhancing spoilage [40] during the rainy season. Different species of yeasts were found in the same manufacturer's yoghurt on different occasions (dry and rainy seasons) suggesting that the contamination was not systematic. Only* Candida kruzei/inconspicua* was found in at least one sample from all the different groups (except those from Dschang). It was found in 7 out of 14 (dry season) and in 6 out of 13 samples containing yeasts (Table 6). Several species of* Candida *have been reported as contaminant in yoghurt [83, 84]. They mainly involve deterioration [13, 14] and also responsible for off-flavours and loss of texture quality due to gas production during lactose assimilation [19].* C. albicans *is a member of the normal flora of the skin and oral cavity; its presence in the samples may be due to high sugar content of yoghurt [72]. With the presence of these yeasts species, these yoghurts may expose their consumers to possible risk of fungal infections, of which candidiasis is the most deleterious and life threatening [85]. Some reports suggest that the public health significance of yeast contaminants in foods is negligible as few known pathogenic yeasts, such as* Candida albicans* and* Cryptococcus neoformans, *are not transmitted through foods [14, 86]. However, the public health safety of yeasts in foods may need some rethinking as there have been occasional reports of gastroenteritis from foods, wherein yeasts were suspected to be the causative agent [87]. Some representatives of the genus* Rhodotorula *cause staining and give a bitter taste to the products. Warmer weather, inadequate refrigeration, and improper storage are the principal causes of higher levels of contamination, increased diversity, and change in yeast mycoflora [40]. Also yeast species mainly representatives of the genera* Candida *and* Rhodotorula *have been known to decrease the quality of dairy products by lactose assimilation [84, 88, 89]. Spoilage of yoghurts by yeasts has emerged as a major problem in the dairy industry [72, 78]. Interestingly, 7 (12.72%) of the yeast species* (Kloeckera *sp.*, Trichosporon asahii, Cryptococcus humicola, and Kodamaea ohmeri)* could utilize lactose which is an important technological property in milk fermentation. The yeast species identified in the present study might have originated from the ingredients used as well as processing equipment that might not had been properly cleaned and sanitized. Starter cultures of lactic acid bacteria used to ferment the yoghurt are another potential source of yeast contamination. This suggests that overall improved and high quality of hygienic precautions should be adopted to avoid contamination especially during the production of yoghurt.

## 4. Conclusion

In view of the above results, locally made yoghurt samples obtained from Bamenda, Dschang, Bafoussam, and even some of the commercial samples constitute a high risk of health hazard to the consumers especially during the rainy season. The findings of this study warrant the need to undertake safety measures to avoid potential threats and apply educational programs for dairy products producers about the risk of contamination, prevention, and reduction of these pathogens from the yoghurt. Nevertheless, strict hygienic measures need to be applied during production, storage, and distribution of the yoghurts. License given to small dairy producers must be issued after the assurance of a minimum level of GMP. Periodical inspection must be done by specialists on the production sites to ensure that this minimum level of GMP is respected and sanctions should be applied where necessary. Moreover, regulation of small scale (locally made) yoghurt production in Cameroon should be a part of a strategy to enhance production of safe and high quality yoghurts. Finally, branded yoghurts are supposed to be products of high standard but in this case these products are not safe for consumption (except BB). There is equal need for a HACCP (Hazard Analysis and Critical Control Points) program for the production of yoghurt in Cameroon.

## Figures and Tables

**Table 1:** The microbial quality of yoghurt samples as a function of production area and producer during the dry season (I).

Samples	Location	Microbial colony counts (log 10 CFU/ml)				
		Total bacterial count	Coliform counts	Total fungal counts	Yeast counts	Mould counts
V	Dschang	11.63 ± 0.10^ij^	4.33 ± 0.16^defg^	4.27 ± 0.13^c^	4.02 ± 0.21^bc^	3.56 ± 0.24^cdef^
P	Dschang	9.28 ± 0.00^cd^	5.12 ± 0.09^g^	6.12 ± 0.13^i^	4.97 ± 0.11^c^	4.26 ± 0.11^f^
G	Dschang	11.04 ± 0.11^f^	4.29 ± 0.20^defg^	5.56 ± 0.24^h^	3.73 ± 3.23^b^	1.10 ± 1.90^ab^
NR	Bamenda	11.56 ± 0.02^ij^	4.40 ± 0.03^efg^	4.63 ± 0.07^ef^	4.47 ± 0.19^bc^	4.05 ± 0.18^def^
S	Bamenda	11.78 ± 0.02^k^	4.00 ± 0.19^cdef^	4.25 ± 0.07^bc^	4.17 ± 0.11^bc^	2.40 ± 0.17^bcde^
D	Bamenda	9.62 ± 0.02^e^	3.88 ± 0.50^cde^	4.34 ± 0.13^cd^	4.27 ± 0.09^bc^	2.35 ± 2.05^bcd^
MR	Bamenda	8.80 ± 0.06^a^	4.73 ± 0.09^fgh^	4.91 ± 0.09^g^	4.87 ± 0.08^bc^	3.60 ± 0.30^cdef^
MB	Bamenda	9.40 ± 0.17^d^	3.53 ± 0.40^cd^	5.04 ± 0.12^g^	4.94 ± 0.15^bc^	4.11 ± 0.13^ef^
PA	Bamenda	9.16 ± 0.02^bc^	3.30 ± 0.00^c^	0.00 ± 0.00^a^	0.00 ± 0.00^a^	0.00 ± 0.00^a^
SY	Bamenda	9.17 ± 0.07^bc^	4.82 ± 0.16^fgh^	4.93 ± 0.06^g^	4.91 ± 0.07^bc^	2.40 ± 2.07^bcde^
PV	Bamenda	9.15 ± 0.05^b^	3.53 ± 0.40^cd^	4.43 ± 0.03^cde^	4.38 ± 0.15^bc^	2.20 ± 1.90^bc^
T	Bafoussam	11.36 ± 0.14^h^	6.90 ± 0.08^i^	4.54 ± 0.13^de^	4.54 ± 0.13^bc^	0.00 ± 0.00^a^
Ce	Bafoussam	11.18 ± 0.04^g^	1.25 ± 2.18^b^	4.02 ± 0.10^b^	4.02 ± 0.10^bc^	0.00 ± 0.00^a^
C	Bafoussam	11.30 ± 0.08^h^	5.37 ± 0.04^h^	4.83 ± 0.07^fg^	4.63 ± 0.13^bc^	3.50 ± 0.17^cdef^
K	Bafoussam	11.30 ± 0.05^h^	4.31 ± 0.19^defg^	4.61 ± 0.04^ef^	4.11 ± 0.35^bc^	2.53 ± 2.20^bcdef^

AA	Commercial 1	9.54 ± 0.05^e^	4.57 ± 0.31^efgh^	4.84 ± 0.49^fg^	4.84 ± 0.49^bc^	0.00 ± 0.00^a^
BB	Commercial 2	11.35 ± 0.03^h^	0.00 ± 0.00^a^	0.00 ± 0.00^a^	0.00 ± 0.00^a^	0.00 ± 0.00^a^
CC	Commercial 3	11.48 ± 0.11^i^	4.81 ± 0.05^fgh^	4.65 ± 0.10^ef^	4.65 ± 0.10^bc^	0.00 ± 0.00^a^

Values are mean ± SD of 3 determinants. Along the columns, values with the same letter (a, b, c, d, e, f, and g) are not significantly different (*p* > 0.05).

**Table 2:** The microbial quality of yoghurt samples as a function of production area and producer during the rainy season (II).

Samples	Location	Microbial colony counts (log 10 CFU/ml)				
		Total bacterial count	Coliform counts	Total fungal counts	Yeast counts	Mould counts
P	Dschang	8.70 ± 0.09^a^	5.55 ± 0.07^hi^	5.02 ± 0.05^h^	4.87 ± 0.10^fg^	4.30 ± 0.21^e^
G	Dschang	11.55 ± 0.06^h^	4.49 ± 0.35^cdef^	4.06 ± 0.20^c^	4.05 ± 0.18^c^	0.00 ± 0.00^a^
SY	Bamenda	11.64 ± 0.02^hi^	4.43 ± 0.08^cdef^	4.57 ± 0.06^def^	4.39 ± 0.02^d^	3.96 ± 0.21^de^
NR	Bamenda	9.17 ± 0.13^b^	3.87 ± 0.33^c^	4.54 ± 0.05^de^	4.06 ± 0.15^c^	4.43 ± 0.01^e^
PV	Bamenda	11.18 ± 0.05^f^	4.10 ± 0.30^cd^	3.94 ± 0.18^bc^	3.66 ± 0.39^b^	3.40 ± 0.17^d^
PA	Bamenda	11.23 ± 0.04^fg^	5.46 ± 0.27^ghi^	0.00 ± 0.00^a^	0.00 ± 0.00^a^	0.00 ± 0.00^a^
D	Bamenda	11.03 ± 0.11^e^	3.76 ± 0.40^c^	4.46 ± 0.10^d^	4.34 ± 0.12^d^	3.77 ± 0.00^de^
MB	Bamenda	9.55 ± 0.04^c^	3.84 ± 0.27^c^	3.73 ± 0.37^b^	3.70 ± 0.34^b^	2.35 ± 2.05^c^
S	Bamenda	11.06 ± 0.04^e^	4.27 ± 0.18^cde^	4.08 ± 0.15^c^	4.08 ± 0.15^c^	0.00 ± 0.00^a^
MR	Bamenda	11.73 ± 0.01^i^	5.51 ± 0.03^hi^	4.41 ± 0.06^d^	4.41 ± 0.06^d^	0.00 ± 0.00^a^
T	Bafoussam	11.19 ± 0.04^f^	4.76 ± 0.15^defg^	5.05 ± 0.02^h^	5.05 ± 0.02^g^	0.00 ± 0.00^a^
Ce	Bafoussam	11.75 ± 0.03^i^	5.10 ± 0.02^fghi^	4.92 ± 0.08^gh^	4.92 ± 0.08^fg^	0.00 ± 0.00^a^
C	Bafoussam	11.02 ± 0.08^e^	0.00 ± 0.00^a^	4.61 ± 0.09^def^	4.49 ± 0.31^de^	1.30 ± 2.25^b^
K	Bafoussam	11.27 ± 0.02^fg^	1.20 ± 2.07^b^	4.52 ± 0.11^d^	4.52 ± 0.11^de^	0.00 ± 0.00^a^

AA	Commercial 1	11.02 ± 0.17^e^	4.85 ± 0.04^efgh^	4.50 ± 0.18^d^	4.50 ± 0.18^de^	0.00 ± 0.00^a^
BB	Commercial 2	11.34 ± 0.06^g^	0.00 ± 0.00^a^	0.00 ± 0.00^a^	0.00 ± 0.00^a^	0.00 ± 0.00^a^
CC	Commercial 3	10.34 ± 0.12^d^	4.34 ± 0.09^cde^	4.77 ± 0.17^efg^	4.77 ± 0.17^ef^	0.00 ± 0.00^a^

Values are mean ± SD of 3 determinants. Along the columns, values with the same letter (a, b, c, d, e, f, g, and h) are not significantly different (*p* > 0.05).

**Table 3:** Enterobacteriaceae found in yoghurts samples from two different batches as a function of sample group and season.

Place	Producers	Enterobacteriaceae species/season				Total number of each Enterobacteriaceae from each group/season	
		I	% of identification	II	% of identification	I	II
Bamenda	NR	*Pantoea *sp.* 1*,	79.30	*Pantoea *sp.* 3*	75.89	*Pantoea *sp.* 1 *(2), *Pantoea *sp.* 4* (1), *Raoultella ornithinolytica *(1), *Shigella *sp. (2), *Serratia plymuthica* (1), *Burkholderia cepacia *(1),* Providencia stuartii *(2), *Providencia alcalifaciens/rustigianii* (1), *Pseudomonas oryzihabitans* (1)	*Pantoea *sp.* 1 *(1), *Pantoea *sp.* 3 *(3) *Pantoea *sp.* 4* (1), * Enterobacter cloacae *(2), *Shigella *sp. (3), *Serratia plymuthica *(1), *Burkholderia cepacia* (1), *Providencia stuartii *(2),* Moellerella wisconsensis *(1)
		*Enterobacter cloacae*	95.27				
	MR	*Raoultella ornithinolytica*,	91.05	*Pantoea *sp.* 3*,	94.90		
		*Enterobacter cloacae*,	95.27	*Serratia plymuthica*,	91.39		
		*Shigella *sp.	70.17	*Shigella *sp.,	66.01		
				*Burkholderia cepacia *	81.30		
	D	*Serratia plymuthica*	91.39	*Shigella *sp.,	69.04		
				*Pantoea *sp.* 4*	75.58		
	PV	*Shigella *sp.,	69.04	*Shigella *sp.	69.04		
		*Providencia stuartii*,	86.08				
		*Providencia alcalifaciens/rustigianii *	61.94				
	SY	*Pantoea *sp.* 4*	86.48	*Providencia stuartii *	91.13		
		*Pantoea *sp.* 1*	80.43	*Pantoea *sp.* 3*	75.89		
	S	*Pseudomonas oryzihabitans*	82.77	*Moellerella wisconsensis*	89.38		
		*Burkholderia cepacia*	81.30	*Providencia stuartii*	71.56		
	MB	*Providencia stuartii*	86.08	*Pantoea *sp.* 1*	73.72		

Dschang	P	*Moellerella wisconsensis*	88.87	*Klebsiella pneumonia *ssp.* pneumonia*	97.69	*Moellerella wisconsensis *(1), *Klebsiella pneumonia *ssp.* pneumonia* (2), *Pasteurella pneumotropica/Mannheimia haemolytica* (1), *Pantoea *sp.* 1 *(2), *Providencia rettgeri* (1), *Ochrobactrum anthropi* (1),* Enterobacter cloacae* (1),* Pseudomonas oryzihabitans* (1)	*Klebsiella pneumonia *ssp.* pneumonia* (3), * Providencia rettgeri* (1)
		*Pasteurella pneumotropica/Mannheimia haemolytica*	70.36	*Klebsiella pneumonia *ssp.* pneumonia*	98.88		
		*Pantoea *sp.* 1*	73.72	*Providencia rettgeri*	100		
	V	*Enterobacter cloacae*	81.23	/	/		
		*Pseudomonas oryzihabitans *	85.72				
	G	*Klebsiella pneumonia *ssp.* pneumonia*	97.69	*Klebsiella pneumonia *ssp.* pneumonia*	97.69		
		*Klebsiella pneumonia *ssp.* pneumonia*	97.69				
		*Providencia rettgeri*	100				
		*Ochrobactrum anthropi*	92.49				
		*Pantoea *sp.* 1*	79.30				

Bafoussam	T	*Klebsiella pneumonia *ssp.* pneumonia*	98.85	*Serratia plymuthica *	91.39	*Klebsiella pneumonia *ssp.* pneumonia* (4), *Enterobacter aerogenes* (2), *Serratia plymuthica* (1),* Pantoea *sp.* 1* (6), *Burkholderia cepacia *(1), *Shigella *sp. (1)	*Serratia plymuthica* (2), *Citrobacter freundii* (2), *Klebsiella oxytoca* (1), *Escherichia coli 1* (3), *Pantoea *sp.* 1 *(3), *Providencia stuartii *(1)
		*Klebsiella pneumonia *ssp.* pneumonia*	97.69	*Citrobacter freundii *	99.99		
		*Klebsiella pneumonia *ssp.* pneumonia*	98.01	*Citrobacter freundii *	99.99		
		*Enterobacter aerogenes*	78.96	*Klebsiella oxytoca *	98.46		
				*Serratia plymuthica*	86.82		
	Ce	*Pantoea *sp.* 1*	90.19	*Escherichia coli 1*	99.84		
		*Pantoea *sp.* 1*	90.49	*Escherichia coli 1*	92.99		
				*Escherichia coli 1*	99.02		
				*Providencia stuartii*	91.13		
	C	*Pantoea *sp.* 1*	80.43	*Pantoea *sp.* 1*	90.19		
		*Pantoea *sp.* 1*	90.49				
		*Serratia plymuthica*	91.39				
		*Burkholderia cepacia*	71.24				
	K	*Shigella *sp.	69.04	*Pantoea *sp.* 1*	90.75		
		*Pantoea *sp.* 1*	90.75	*Pantoea *sp.* 1*	81.83		
		*Pantoea *sp.* 1*	90.75				

Commercial	AA	*Enterobacter aerogenes*	83.44	*Klebsiella pneumonia *ssp.* pneumonia*	98.67	*Enterobacter aerogenes* (1), *Klebsiella pneumonia *ssp.* pneumonia* (1), *Shigella *sp. (1)	*Klebsiella pneumonia *ssp.* pneumonia* (1), *Pantoea *sp.* 1 *(1),* Proteus penneri* (1)
		*Klebsiella pneumonia *ssp.* pneumonia*	98.67				
	CC	*Shigella *sp.	83.19	*Pantoea *sp.* 1 *	83.19		
				*Proteus penneri*	77.49		

^1^The letters I and II refer to batches of the dry and rainy seasons, respectively. ^2^The sixteen manufacturers/producers are designated as NR, MR, D, PV, SY, S, MB, P, V, G, T, Ce, C, K, AA, and CC. ^3^ Numbers in parentheses are the number of isolates identified as that species.

**Table 4:** The distribution of Enterobacteriaceae as a function of sample group and season.

Yoghurt samples/season									Frequency of occurrence	% of occurrence	Frequency of occurrence	% of occurrence
Gram negative bacteria isolated from yoghurt	Bamenda		Dschang		Bafoussam		Commercial					
	(I)	(II)	(I)	(II)	(I)	(II)	(I)	(II)	(I)	(I)	(II)	(II)
*Pantoea *sp.* 1*	+	+	+	−	+	+	−	+	3	75	3	75
*Pantoea *sp.* 3*	−	+	−	−	−	−	−	−	0	00	1	25
*Pantoea *sp.* 4*	+	+	−	−	−	−	−	−	1	25	1	25
*Raoultella ornithinolytica*	+	−	−	−	−	−	−	−	1	25	0	00
*Shigella *sp.	+	+	−	−	+	−	+	−	3	75	1	25
*Serratia plymuthica*	+	+	−	−	+	+	−	−	2	50	2	50
*Burkholderia cepacia*	+	+	−	−	+	−	−	−	2	50	1	25
*Providencia stuartii*	+	+	−	−	−	+	−	−	1	25	2	50
*Providencia rettgeri*	−	−	+	+	−	−	−	−	1	25	1	25
*Providencia alcalifaciens/rustigianii*	+	−	−	−	−	−	−	−	1	25	0	00
*Pseudomonas oryzihabitans*	+	−	+	−	−	−	−	−	2	50	0	00
*Moellerella wisconsensis*	−	+	+	−	−	−	−	−	1	25	1	25
*Klebsiella pneumonia *ssp.* pneumonia*	−	−	+	+	+	−	+	+	3	75	2	50
*Klebsiella oxytoca*	−	−	−	−	−	+	−	−	0	00	1	25
*Pasteurella pneumotropica/Mannheimia haemolytica*	−	−	+	−	−	−	−	−	1	25	0	00
*Ochrobactrum anthropi*	−	−	+	−	−	−	−	−	1	25	0	00
*Enterobacter aerogenes*	−	−	−	−	+	−	+	−	2	50	0	00
*Enterobacter cloacae*	−	+	+	−	−	−	−	−	1	25	1	25
*Citrobacter freundii*	−	−	−	−	−	+	−	−	0	00	1	25
*Escherichia coli 1*	−	−	−	−	−	+	−	−	0	00	1	25
*Proteus penneri*	−	−	−	−	−	−	−	+	0	00	1	25

^1^The letters I and II refer to batches of the dry and rainy seasons, respectively; + = positive, − = negative.

**Table 5:** Yeast species in yoghurts samples from two different batches as a function of sample group and season.

Place	Producers	Yeast species/season				Total number of each yeast from each group/season	
		I	% of identification	II	% of identification	I	II
Bamenda	NR	*Candida kruzei/inconspicua*	95.33	*Rhodotorula mucilaginosa 1*	99.95	*Candida kruzei/inconspicua* (5), *Candida dubliniensis* (2),* Candida zeylanoides* (3), *Trichosporon asahii* (1)	*Candida kruzei/inconspicua* (4), *Candida zeylanoides* (8), *Candida boidinii* (2), *Rhodotorula mucilaginosa 1* (1), *Rhodotorula mucilaginosa 2* (1), *Stephanoascus ciferrii* (1)
		*Candida dubliniensis*	100	*Candida kruzei/inconspicua*	95.33		
				*Candida kruzei/inconspicua*	64.12		
				*Candida boidinii*	93.38		
	MR	*Trichosporon asahii*	100	*Candida zeylanoides*	97.96		
		*Candida zeylanoides*	99.94	*Candida kruzei/inconspicua*	95.33		
		*Candida kruzei/inconspicua*	95.33				
	PV	*Candida kruzei/inconspicua*	95.33	*Rhodotorula mucilaginosa 2*	99.89		
				*Candida zeylanoides*	99.94		
				*Candida zeylanoides*	97.96		
	SY	*Candida kruzei/inconspicua*	95.14	*Candida zeylanoides*	99.94		
		*Candida zeylanoides*	99.98	*Stephanoascus ciferrii*	84.98		
				*Candida zeylanoides*	95.58		
				*Candida kruzei/inconspicua*	95.33		
	MB	*Candida zeylanoides*	99.98	*Candida zeylanoides*	99.94		
		*Candida kruzei/inconspicua*	61.36	*Candida zeylanoides*	99.98		
		*Candida dubliniensis*	100	*Candida zeylanoides*	99.54		
				*Candida boidinii*	93.38		

Dschang	P	*Candida zeylanoides*	99.94	*Kodamaea ohmeri*	81.82	*Candida zeylanoides* (3), *Pichia angusta* (1) *Candida lusitaniae* (1), *Kodamaea ohmeri* (1), *Trichosporon asahii* (1)	*Kodamaea ohmeri* (1), *Trichosporon asahii* (1)
		*Pichia angusta*	94.91				
		*Candida zeylanoides*	99.98				
	V	*Candida lusitaniae*	82.17	*/*	/		
		*Kodamaea ohmeri*	74.44				
		*Trichosporon asahii*	99.98				
	G	*Candida zeylanoides*	99.94	*Trichosporon asahii*	99.98		

Bafoussam	T	*Cryptococcus humicola*	76.21	*Candida dubliniensis*	100	*Candida kruzei/inconspicua* (2), *Candida zeylanoides* (1), *Candida albicans 2* (1), *Cryptococcus humicola* (1)	*Candida kruzei/inconspicua* (1), *Candida dubliniensis* (2), *Candida albicans 1* (1), *Candida lusitaniae* (1), *Cryptococcus laurentii* (1), *Kloeckera *sp. (1)
	Ce	*Candida kruzei/inconspicua*	89.93	*Candida kruzei/inconspicua*	89.93		
				*Candida albicans 1*	96.06		
	C	*Candida kruzei/inconspicua*	95.33	*Cryptococcus laurentii*	100		
		*Candida zeylanoides*	99.94	*Kloeckera sp*	65.10		
	K	*Candida albicans 2*	66.76	*Candida dubliniensis*	100		
				*Candida lusitaniae*	62.97		

Commercial	AA	*Candida kruzei/inconspicua*	95.14	*Candida lusitaniae *	97.88	*Candida kruzei/inconspicua* (1), *Candida dubliniensis* (1),	*Candida lusitaniae* (1), *Candida kruzei/inconspicua* (1), *Candida dubliniensis* (1), *Stephanoascus ciferrii* (1)
				*Candida kruzei/inconspicua *	95.14		
				*Stephanoascus ciferrii*	62.16		
	CC	*Candida dubliniensis*	100	*Candida dubliniensis*	100		

^1^The letters I and II refer to batches of the dry and rainy seasons, respectively. ^2^Numbers in parentheses are the number of isolates identified as that species.

**Table 6:** The distribution of yeast species as a function of sample group and season.

Yoghurt samples/season									Frequency of occurrence	% of occurrence	Frequency of occurrence	% of occurrence
Yeast species isolated from yoghurt	Bamenda		Dschang		Bafoussam		Commercial					
	(I)	(II)	(I)	(II)	(I)	(II)	(I)	(II)	(I)	(I)	(II)	(II)
*Candida kruzei/inconspicua*	+	+	−	−	+	+	+	+	3	75	3	75
*Candida boidinii*	−	+	−	−	−	−	−	−	0	00	1	25
*Candida dubliniensis*	+	−	−	−	−	+	+	+	2	50	2	50
*Candida zeylanoides*	+	+	+	−	+	−	−	−	3	75	1	25
*Candida lusitaniae*	−	−	+	−	−	+	−	+	1	25	3	50
*Candida albicans 1*	−	−	−	−	−	+	−	−	0	00	1	25
*Candida albicans 2*	−	−	−	−	+	−	−	−	1	25	0	00
*Rhodotorula mucilaginosa 1*	−	+	−	−	−	−	−	−	0	00	1	25
*Rhodotorula mucilaginosa 2*	−	+	−	−	−	−	−	−	0	00	1	25
*Stephanoascus ciferrii*	−	+	−	−	−	−	−	+	0	00	2	50
*Trichosporon asahii*	+	−	+	+	−	−	−	−	2	50	1	25
*Kodamaea ohmeri*	−	−	+	+	−	−	−	−	1	25	1	25
*Pichia angusta*	−	−	+	−	−	−	−	−	1	25	0	00
*Cryptococcus laurentii*	−	−	−	−	−	+	−	−	0	00	1	25
*Cryptococcus humicola*	−	−	−	−	+	−	−	−	1	25	0	00
*Kloeckera *sp.	−	−	−	−	−	+	−	−	0	00	1	25

^1^The letters I and II refer to batches of the dry and rainy seasons, respectively; + = positive, − = negative.
